# Improving Congestion Control of TCP for Constrained IoT Networks

**DOI:** 10.3390/s20174774

**Published:** 2020-08-24

**Authors:** Chansook Lim

**Affiliations:** Department of Software & Communications Engineering, Hongik University, Sejong 30016, Korea; chansooklim@hongik.ac.kr; Tel.: +82-44-860-2549

**Keywords:** TCP, IoT network, congestion control, retransmission timer

## Abstract

For smooth integration with middleboxes on the Internet, TCP (Transmission Control Protocol) is favorably considered as a transport-layer protocol for IoT (Internet of Things) networks. In constrained networks, TCP tends to perform well with a small window size. For example, the uIP (micro IP) TCP/IP stack sets the TCP window size to one segment by default. In such a case, managing the retransmission timer is a primary approach to congestion control. In this paper, we examine the congestion control mechanism of TCP in the uIP stack using grid topology networks. In the preliminary test using the Cooja network simulator, the results show that the original uIP TCP causes lots of retransmissions when a radio duty cycling mechanism such as ContikiMAC is used. One main reason is that, once retransmission is deemed to be necessary, the original uIP TCP sets the retransmission timer based on the fixed RTO (retransmission timeout) before performing a retransmission. Since ContikiMAC may cause large RTT (round-trip time) variations due to the hidden terminal problem, the retransmission timer based on the fixed RTO value may cause lots of retransmissions. To address the problem, we propose a new scheme for managing the retransmission timer which adopts the notion of weak RTT estimation of CoCoA, exponential backoffs with variable limits, and dithering. Simulation results show that our proposed scheme reduces retransmissions while enhancing throughput and fairness when an RDC (radio duty cycling) mechanism is used.

## 1. Introduction

TCP, which is a de facto transport layer protocol for the Internet, was not considered a suitable protocol for wireless sensor networks which enable IoT [1,2]. However, protocol standardization for IoT resulted in TCP emerging as a major candidate transport protocol, even for constrained IoT networks, because TCP is better than UDP (User Datagram Protocol) for integration with the existing network infrastructures where middleboxes such as firewalls or NATs (Network Address Translation devices) may block UDP packets. In Reference [1], Gomez et al. stated that the recent industry and standardization tendencies such as the Constrained Application Protocol (CoAP) over TCP, HTTP (Hypertext Transport Protocol) optimized for IoT, and messaging protocols such as the Message Queuing Telemetry Transport (MQTT) and the Advanced Message Queuing Protocol (AMQP) suggest that TCP may gain extensive support in IoT scenarios. Regarding the historically claimed issues of TCP in IoT scenarios, they argue that most of the issues are found in well-accepted IoT end-to-end reliability mechanisms, can be solved, or are not actual issues.

The major challenge of designing a protocol for constrained networks is how to make it work well under severe resource constraints such as limited battery and computing power, small memory, or insufficient wireless bandwidth and ability to communicate [3]. To reduce memory usage, for example, TCP of the uIP stack [4,5] does not implement a sliding window for sending and receiving data and limits the number of outstanding segments to one. Several other embedded TCP implementations including BLIP (the Berkeley Low-power IP) stack [6] in TinyOS [7] and GNRC (the Generic Network Stack) [8] in RIOT OS [9] also keep the TCP window size of one segment [10,11,12]. However, this does not mean that these TCP implementations do not need any congestion control mechanism. Since periodic reporting by every node in scenarios such as continuous monitoring may cause congestion around the sink, TCP still needs to control congestion. In this case, managing the retransmission timer is a primary approach to congestion control. The optimal mechanism for setting the retransmission timer value will reduce retransmissions while maintaining high throughput. Reducing TCP retransmissions in wireless sensor networks is very important because end-to-end retransmission consumes much more energy than hop-by-hop retransmission. 

Radio duty cycling (RDC) is a major tool which is considered to save battery power in wireless sensor networks. However, despite a number of prior works on TCP for wireless sensor networks [10,13,14,15,16], few studies considered RDC. TCP is required to work well regardless of whether an RDC mechanism runs underneath. We examined TCP performance in constrained networks with RDC enabled. In the preliminary test using the Cooja network simulator, we found that uIP TCP causes lots of retransmissions when ContikiMAC [17], which is the default radio duty cycling mechanism in the Contiki OS, is enabled. uIP TCP’s mechanism for managing the retransmission timer explains a considerable part of the problem. uIP TCP sets the retransmission timer based on RTT measurements for first transmissions. However, for retransmissions, it sets the retransmission timer based on the fixed retransmission timeout (RTO) of 3 s. Since ContikiMAC may cause large RTT variations due to the hidden terminal problem, the retransmission timer based on the fixed RTO value may cause lots of retransmissions, which could be reduced with the estimated RTO based on RTT measurements. This motivated us to design a scheme that enhances uIP TCP’s performance in networks with RDC enabled.

In devising a mechanism for managing the retransmission timer, we note that CoAP [18], which is an application protocol for constrained networks, also usually limits the number of outstanding messages to one. Congestion control relying only on RTO management was actively studied in the context of CoAP over UDP. The CoAP Simple Congestion Control/Advanced (CoCoA) [19,20] is one of the well-known schemes to enhance congestion control for CoAP. We propose a scheme that enhances uIP TCP’s performance by employing the notions introduced by CoCoA such as weak RTT and backoff with variability.

Some might argue that in-network congestion control is better than per-flow end-to-end congestion control in IoT networks. In-network congestion control is effective because network devices can use the information which is not available to end systems. Therefore, to improve congestion control and load balancing at the IPv6 Routing Protocol for Low-Power and Lossy Networks (RPL) layer, numerous RPL schemes, including ORPL (Opportunistic RPL), QU-RPL (Queue utilization based RPL), PC-RPL (Power-controlled RPL), and BRPL (Backpressure RPL), were proposed [21,22,23,24]. However, to prevent the sources from overwhelming the network, we also need an end-to-end congestion control mechanism that makes the sources control the sending rates. Both in-network approaches and end-to-end approaches can complement each other.

Our main contribution can be summarized as follows:

- We investigate the effect of RTT and RTO estimation on uIP TCP performance in constrained networks.
- We examine how to adopt, for uIP TCP, the notion of weak RTT and backoff with variability, which CoCoA uses for congestion control in CoAP.
- We propose a new mechanism for managing the retransmission timer to enhance TCP performance in constrained networks with RDC enabled.

The rest of the paper is organized as follows: Section 2 provides the related work; Section 3 provides a brief overview of uIP TCP and CoCoA; Section 4 discusses the preliminary test results; Section 5 presents our proposed scheme; Section 6 discusses performance evaluation. Lastly, in Section 7, we draw conclusions.

## 2. Related Work

We summarize the recent studies related to our work in two categories: studies on TCP for IoT networks and studies on congestion control for CoAP. 

### 2.1. Studies on TCP for IoT Networks

Hurni et al. explored how to optimize TCP performance by examining the effect of distributed caching and local retransmission strategies where each intermediate node caches TCP segments and retransmits a segment whose ACK (acknowledgement) is considerably delayed, based on RTT estimation [14]. They integrated their implementation named the caching and congestion control (cctrl) module into the uIP stack of Contiki OS and tested it using different radio duty cycling MAC (medium access control) protocols in a real testbed comprising seven TelosB motes. Experimental results showed that the cctrl module increased TCP throughput in many of the examined configurations, but its performance depended heavily on the underlying RDC MAC protocols.

In Reference [10], Kim et al. conducted an experimental study on performance of TCP over RPL in an IPv6-based testbed which is a low-power and lossy network (LLN) consisting of 30 TelosB devices using the TinyOS BLIP stack, one LBR (LoWPAN border router), and one Linux-based server. They found that TCP incurred significant throughput unfairness among nodes in multihop LLNs, and that RPL’s inability to consider traffic load balancing may adversely affect TCP performance.

To address the TCP fairness problem among LLN endpoints, Park and Paek proposed TAiM (TCP assistant in the middle) [16], which intervenes in the middle of TCP communication only at LBR and manipulates RTT of the passing flows. TAiM buffers packets and intentionally adds time delay before every forwarding, so that a flow with low throughput can have shorter delay, whereas a flow with high throughput may have longer delay. Experimental results using the BLIP stack showed that TAiM improved TCP fairness while maintaining total throughput.

In Reference [1], Gomez et al. provided recommendations for lightweight TCP implementation and suitable operation in IoT scenarios. In particular, as far as an RTO algorithm is concerned, they recommended using CoCoA for TCP.

As powerful low-power embedded devices with more processing power and larger memory space emerge, Kumar et al. showed that a full-scale TCP can fit well within CPU and memory constraints of modern wireless sensor network platforms by implementing a full-scale TCP, called TCPlp [12,15], leveraging the full-featured TCP in FreeBSD OS. 

### 2.2. Studies on Congestion Control for CoAP

In devising a congestion control mechanism of TCP for constrained networks, it is worthwhile to refer to CoAP, which was defined by the IETF (Internet Engineering Task Force) CoRE (Constrained RESTful Environments) Working Group. For CoAP running over UDP, lightweight reliability is supported using confirmable (CON) messages and ACK messages. If the sender does not receive any ACK message acknowledging a CON message before the retransmission timer expires, the CON message is retransmitted as long as the retransmission counter does not exceed MAX_RETRANSMIT, which is usually set to four. The basic congestion control of CoAP is carried out by setting RTO without measuring RTT and by limiting the number of outstanding CON messages. For each new CON message, the initial RTO is randomly chosen between 1 × ACK_TIMEOUT and ACK_RANDOM_FACTOR × ACK_TIMEOUT. Since the default values of ACK_TIMEOUT and ACK_RANDOM_FACTOR are 2 s and 1.5, respectively, RTO is chosen randomly between 2 and 3 s by default. CoAP employs a binary exponential backoff mechanism which doubles RTO whenever a timeout occurs.

Among numerous efforts to enhance the basic congestion control of CoAP, the best-known congestion control mechanism for CoAP is CoCoA [19,20]. The key features of CoCoA include weak RTO estimator and variable backoff factor (VBF). The weak RTO estimator also uses retransmitted segments when updating RTT estimates. We describe CoCoA in more detail in Section 3.

CoCoA was examined by a number of studies. In Reference [25], the authors reported that CoCoA can perform significantly worse than default CoAP, especially with bursty traffic and in networks with few clients as a result of an improper selection of the retransmission timeouts.

CoCoA 4-state-strong [26] addresses the performance issue of CoCoA in lossy networks. The authors reported that CoCoA underperformed the default CoAP in highly lossy networks due to inaccurate measurement of the weak RTT estimator and excessive RTO backoff on channel losses. CoCoA 4-state-strong attempts to enhance the accuracy of RTT measurement by using a retransmission ID or by measuring the time from the latest transmission. CoCoA 4-state-strong uses four states to determine the RTO backoff factor and the weight for the overall RTO calculation. Each CoAP transaction starts in state 1. The state transitions according to (re)transmission and acknowledgement. For each transmission, the transaction moves to the next higher state. Conversely, for each acknowledgement, the transaction moves to the next lower state. For a higher state, the backoff factor is set to a higher value. For a large RTO value, a small backoff factor is used. 

Precise congestion control algorithm (pCoCoA) [27] is another scheme to overcome the limitations of CoCoA by enhancing the accuracy of RTT. The authors pointed out that CoCoA may incur many spurious retransmissions at some offered loads close to congestion and that the weak estimator is not effective in adapting to changing traffic loads. pCoCoA eliminates the use of the weak estimator by introducing a CoAP option named the transmission count (TC) option to match each ACK message with the corresponding CON message even when it is a retransmitted CON message. To adapt more effectively to changing traffic load, pCoCoA computes RTO in a similar way to Linux TCP.

It is notable that that several studies including CoCoA 4-state-strong and pCoCoA suggested measuring RTT more accurately by using the (re)transmitted ID or counter option in CoAP messages [26,27]. However, this approach is not suitable for TCP because it is not feasible to use a field in the TCP header for a specific purpose. Since TCP is a widely used transport-layer protocol, the TCP option used for special networks is likely to be easily ignored by the counterpart TCP which is a general implementation.

The early version of CoCoA [28] is slightly different from the current version. Like the current version, the early version adopted the weak estimator and computed the overall RTO estimate, which is an exponentially weighted moving average computed of the strong and the weak estimator. However, unlike the current version, it computed SRTT (smoothed RTT), RTTVAR (RTT variance), and RTO according to RFC (Request for Comments) 6298 [29] and used a binary exponential backoff mechanism instead of using the variable backoff factor. In Reference [30], the authors applied the idea of an Eifel retransmission timer [31] to the early version of CoCoA to prevent making RTO too aggressive or too conservative. The existing estimator gains (i.e., the constant coefficients α and β) used for computing SRTT and RTTVAR can make RTO aggressive. To address the problem, the authors proposed replacing the constant coefficients with a dynamically changing parameter defined as the ratio between the current RTT sample and the RTO value. The authors also pointed out that negative mean deviation can cause undesirable effects; an RTT decrease can increase RTTVAR, thereby increasing RTO as opposed to an indication of the RTT decrease. The authors addressed this problem by skipping the rule of updating RTTVAR when RTT-SRTT is negative. Comparing the proposed scheme of Reference [30] to our proposed scheme, the two schemes are similar in that SRTT, RTTVAR, and RTO are computed using the same algorithm for both the strong and the weak estimator and a binary exponential backoff mechanism is used. However, when taking a weak RTT sample, the scheme of Reference [30] measures the time from the initial transmission, whereas ours measures the time from the latest transmission. Moreover, while ours computes SRTT, RTTVAR, and RTO according to the classical rule defined in RFC 6298, the scheme of Reference [30] computes SRTT and RTTVAR using a more dynamic parameter.

## 3. Overview of uIP TCP and CoCoA

### 3.1. uIP TCP

The uIP TCP/IP stack is an extremely small implementation of the TCP/IP protocol suite that was designed for embedded systems [4]. uIP uses a single global buffer which is large enough to hold one packet of the maximum size. The same global packet buffer is used both for incoming packets and for the TCP/IP headers of outgoing data. When a packet arrives from the network, the device driver places it in the global buffer and calls the TCP/IP stack. To send data, the application passes a pointer to the data, as well as the length of the data, to the stack. The TCP/IP headers are written into the global buffer, and then the device driver sends the headers and the application data out on the network. Since the data are not saved for retransmission, the application has to reproduce the data if retransmission is necessary.

In uIP, the application is invoked in response to events such as data arriving, an incoming connection request, or a poll request from the stack. The main control loop repeatedly checks whether a packet arrived from the network and whether the periodic timer expires. If a packet arrived, the input handler of the TCP/IP stack is invoked. The periodic timer, which expires every 0.5 s in the current implementation, serves several purposes. Firstly, retransmission is driven by the periodic timer. Whenever the periodic timer expires, the retransmission timer for each connection is decremented. When the retransmission timer reaches zero, a retransmission is performed. Secondly, the periodic timer is used to measure RTT. Each time the periodic timer expires, uIP increments a counter for each connection that has unacknowledged data in the network. When an acknowledgement is received, the current value of the counter is recorded as sampled RTT. uIP TCP uses Karn’s algorithm which does not measure RTT for a retransmitted segment. The basic time unit for RTT and RTO is 1 s. Since RTT and RTO estimations are calculated using Van Jacobson’s fast algorithm [32], which relies only on integer operations, the computational overhead is low. The algorithm for setting RTO is as follows:

Err = RTT − SRTT;

SRTT = SRTT + 0.125 × Err;

MDEV = MDEV + 0.25 × (|Err| − MDEV);

RTO = SRTT + 4 × MDEV.

While the maximum number of retransmissions is set to eight by default, the retransmission timer backs off exponentially up to four times with the fixed RTO value of 3 s.

uIP TCP does not implement the sliding window algorithm, which requires a lot of 32-bit operations. Instead, it allows only a single TCP segment per connection to be unacknowledged at any given time. Even though each node generates low-rate traffic via a stop-and-wait protocol, congestion can occur at areas where traffic is concentrated. Hence, uIP TCP needs congestion control, and an RTO algorithm comes into play.

Allowing only one outstanding TCP segment may cause the uIP TCP sender to interact poorly with the delayed acknowledgement mechanism of the receiver. Because the receiver only receives a single segment at a time, it may wait until the delay acknowledgement timer (which is typically 200 ms but can be as high as 500 ms) expires, which limits the maximum possible throughput of the sender.

### 3.2. CoCoA

We briefly describe the main features of CoCoA below.

**Weak RTO estimator.** Karn’s algorithm does not take RTT samples from retransmitted TCP segments in order to estimate RTT accurately. However, to raise the chances of measuring RTT, CoCoA takes an RTT sample even when the ACK is triggered by a retransmitted CON message. The RTO estimator using retransmitted messages is called the weak RTO estimator, which is differentiated from the strong RTO estimator that uses only CON messages acknowledged without retransmissions. CoCoA ignores RTT samples obtained after the third retransmission to avoid taking a much larger RTT estimate than the actual RTT. The strong RTO estimator is calculated as follows:

E_strong_ = SRTT + max(G, 4 × RTTVAR),

RTO = 0.5 × E_strong_ + 0.5 × RTO,

where *G* is the clock granularity. The weak RTO estimator is calculated as follows:

E_weak_ = SRTT + max(G, RTTVAR),

RTO = 0.25 × E_weak_ + 0.75 × RTO.

It is notable that, for the weak estimator, the RTT variance multiplier is set to 1 instead of 4 to avoid increasing RTO excessively, and that a lower weight is assigned to the weak estimator to reduce the effect of inaccurate measurements.

**Variable backoff factor.** Since the weak estimator of CoCoA may have a larger RTT estimate than the actual one, the binary exponential backoff mechanism (BEB) may cause network underutilization. To address this problem, CoCoA uses a variable backoff factor (VBF) as follows:

$$\mathrm{VBF}\left(\mathrm{RTO}\right)=\{\begin{array}{c} 3\\ 2\\ 1.3 \end{array}\;\;\begin{array}{c} \mathrm{if}\;\mathrm{RTO}<a\\ \mathrm{if}\;a\leq \mathrm{RTO}\leq b\\ \mathrm{if}\;\mathrm{RTO}>b \end{array}$$

where *a* and *b* are the thresholds. CoCoA sets *a* and *b* to 1 s and 3 s, respectively.

**RTO aging.** Furthermore, CoCoA uses an RTO aging mechanism to avoid keeping an RTO which is no longer valid. If the RTO is too small or too large and is not updated for a while, CoCoA tries to make the RTO move close to the default value.

## 4. Preliminary Performance Evaluation

### 4.1. Simulation Settings

**Figure 1:**
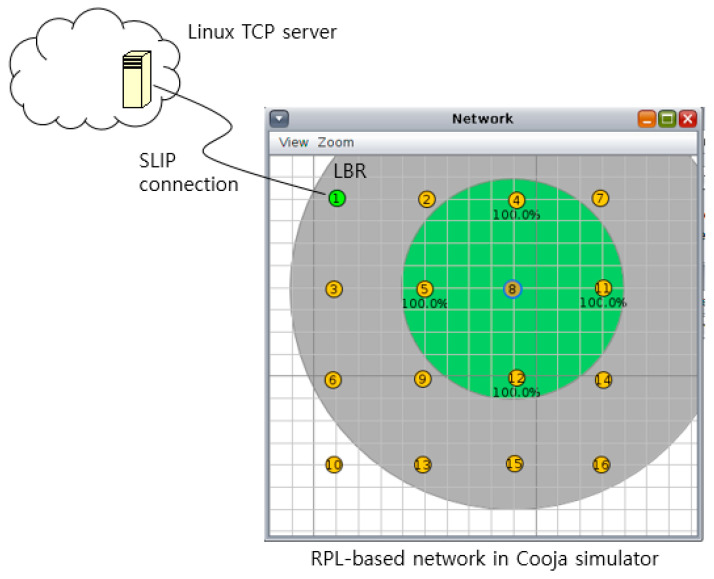
Network configuration for simulation.

We performed a preliminary test using the Cooja network simulator in Contiki 3.x [33]. We simulated two topologies consisting of Sky motes: a 4 × 4 grid topology and a 5 × 5 grid topology. In the 4 × 4 grid topology, the top leftmost node was designated as the LBR, whereas the LBR in the 5 × 5 grid topology was the node in the center. As shown in Figure 1, the LBR was connected to a Linux machine (on a virtual machine) using a slip protocol. While the transmission range and interference range were 50 m and 100 m, respectively, the nodes in a same row or column were 40 m away from each other. Consequently, many nodes suffered severe packet losses due to the hidden terminal problem. Table 1 summarizes the main simulation parameters pertaining to all simulations in this paper. For the preliminary test, we set the random loss rate to 0% in order to focus on congestion; thus, packets were lost only due to packet collision or buffer overflow. The number of packets in the link-layer queue was set to 8 in the preliminary test. 

We wanted to observe the behavior of uIP TCP in networks with an RDC mechanism enabled, which most previous studies on TCP and CoAP for constrained networks did not consider. Simulating both nullRDC and ContikiMAC, we compared the TCP behavior with and without an RDC mechanism enabled. ContikiMAC [17] proposed by Dunkels is the default RDC mechanism in Contiki OS. ContikiMAC is a sender-initiated asynchronous mechanism that uses periodic wake-ups to listen for packet transmissions from neighbors. Nodes sleep most of the time and periodically wake up to check for radio activity. If a packet transmission is detected during a wake-up, the receiver stays awake to receive the packet. When the packet is successfully received, the receiver sends a link layer acknowledgement. A sender repeatedly sends the same packet until it receives a link acknowledgement from the receiver. Once a sender learns the wake-up phase of a receiver via a link layer acknowledgment, the sender can start its successive transmissions to the receiver just before the receiver is expected to be awake. To simulate a typical data collection scenario, each TCP client connects to the TCP server in the Linux machine and sends one segment every 6 s on average. Each TCP segment has a payload with the length of 48 bytes.

At first, we made every node try to connect with the TCP server almost at the same time, but several nodes suffered from TCP connection failures. To avoid TCP connection failures, we made the nodes send connection requests sequentially in a given order with an interval of several seconds so that distant nodes could try connection establishment first. Once a connection is established, each node waits until all the sender nodes can transmit their first TCP data segments almost at the same time. Then, TCP data segments are transmitted for 10 min.

Before obtaining the preliminary test results, we had to fix a problem with handling the retransmission timer. As described in the previous section, the retransmission timer for each connection is decremented every 0.5 s. The problem is that, while the retransmission timer is set in time units of 1 s, it is decremented by one every 0.5 s and, consequently, expires sooner than expected. We fixed this problem by setting the retransmission timer in time units of 0.5 s.

### 4.2. Issues to be Addressed

Through the preliminary test results, we found the below issues in relation to the performance of uIP TCP. 

#### 4.2.1. Using the Fixed RTO Value for Retransmitted Segments

Although uIP TCP measures RTT and estimates RTO based on RTT measurements, the estimated RTO is not used for retransmitted segments. Once retransmission is deemed to be necessary, RTO is fixed at 3 s and backs off exponentially with the number of retransmissions. 

**Figure 2:**
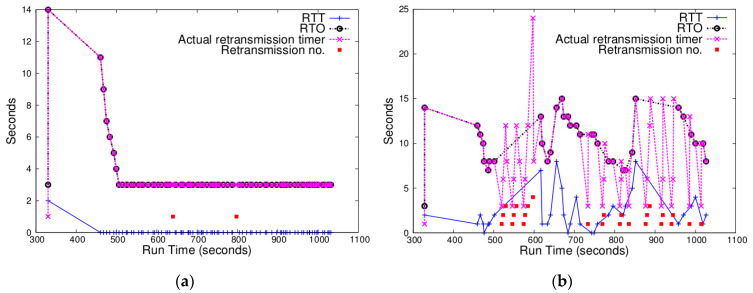
Changes of RTT (blue), RTO (black), actual retransmission timer (pink), and retransmission counter (red) for a node in a 5 × 5 grid topology: (**a**) with NullRDC enabled (i.e., no RDC enabled); (**b**) with ContikiMAC enabled.

Since uIP TCP estimates RTT in time units of 1 s and uses only integer arithmetic operations, a small RTT value is reduced to 0. If RTT measurements keep small, RTO does not exceed 3 s except when the initial large value (16) of RTT mean deviation still has effects on the RTO value at the beginning of a simulation. Hence, the fixed RTO works well. Figure 2a illustrates such a case. When any RDC mechanism is not used, backing off with fixed RTO does not cause many retransmissions because RTT usually remains below 1 s. However, when RTT gets large, using the fixed RTO value may cause lots of retransmissions. Figure 2b shows that, when ContikiMAC is used, RTT fluctuates and many retransmissions occur. In terms of the relation between ContikiMAC and the hidden problem, Hurni et al. stated that, since ContikiMAC lacks preamble strobes in advance of frames, it may more often cause the hidden node problem [14]. In grid topologies, the hidden terminal problem gets more prevalent, which explains why many retransmissions occur when ContikiMAC is used. Note that, even though RTO is updated using the estimated RTT, the actual retransmission timer for a retransmitted segment is set using the fixed RTO of 3 s.

#### 4.2.2. Chances of Measuring RTT

**Figure 3:**
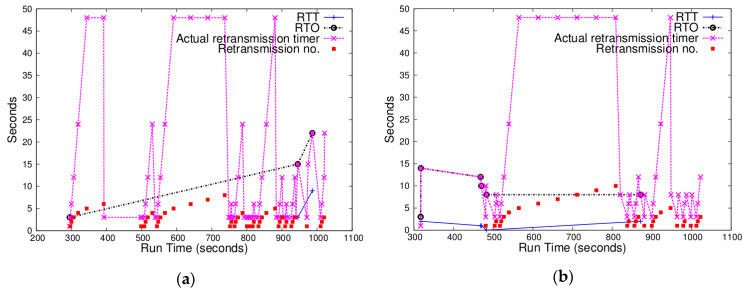
Changes of RTT, RTO, actual retransmission timer, and retransmission counter for some nodes in a 5 × 5 grid topology with ContikiMAC enabled. (**a**) This node had few chances to measure RTT without taking sample RTTs using retransmitted segments. (**b**) In this node, a segment was successfully delivered after 10 retransmissions.

Weak RTT estimation is useful when there are few chances to estimate RTT without taking sample RTTs using retransmitted segments. Figure 3 illustrates the cases where most of the TCP segments after connection establishment were retransmitted. In particular, Figure 3a shows that there were only a couple of RTT measurements which exceeded 3 s. This indicates that utilizing weak RTT estimation might have been able to reduce unnecessary retransmissions.

#### 4.2.3. Limit on the Number of Retransmissions

The implementation of uIP TCP in Cooja sets the maximum number of retransmissions to 8 by default. We note that this limit is much lower than that of Linux. For example, in Linux 2.6+, the default value of tcp_retries2 which limits the number of retransmissions is 15. When the 15th retransmission fails, the connection is deemed broken and the application at the upper layer is notified of the broken connection. With the default limit of 8, we observed many occurrences of delivery failures and even connection failures, particularly when ContikiMAC was used. Hence, for our simulation, we extended the limit to 15. Figure 3b shows that a TCP segment was successfully delivered after 10 retransmissions.

#### 4.2.4. Lack of Dithering

uIP TCP does not have a dithering mechanism. This implies that many of the delivery failures that occur in spite of the maximum retry attempts may be due to a synchronization effect rather than due to congestion. Since each node backs off the retransmission timer based on the fixed RTO value of 3 s, the synchronization effect can be aggravated. We argue that, unlike typical TCP implementations for wired networks, TCP for wireless sensor networks needs to have a dithering mechanism like CoAP.

## 5. Proposed Scheme

As stated above, since uIP TCP and reliable CoAP are similar in that both allow only one outstanding packet by default, the previous studies on congestion control mechanisms of CoAP inspired our proposed scheme. However, we note that uIP TCP and reliable CoAP have different requirements. The most distinctive difference is how to handle a failure of packet delivery after the maximum retransmission attempts. In the case of CoAP, if a message delivery fails even when the number of retransmissions reaches the maximum, the application is notified that the message was not delivered successfully. That is why the delivery ratio is included in the performance evaluation metrics in some CoAP studies. In contrast, it is expected that TCP does not allow delivery failure of a part of the byte stream. As long as the TCP connection is alive, TCP must guarantee that every single segment is delivered to the upper application in order. If a segment fails to be delivered even when the number of retransmissions reaches the maximum number of retrials, a broken network link is notified to the upper layer. Consequently, under the same network conditions, TCP requires that the maximum number of retransmissions be set to a higher value compared to CoAP. However, since retransmission by an end-to-end protocol requires high energy consumption in an energy-restricted network, our main focus is on reducing TCP retransmissions without decreasing throughput. 

As discussed in the previous section, an RDC mechanism may cause large RTT fluctuation, which may lead to many retransmissions. While the purpose of RDC is energy efficiency, inducing many retransmissions can nullify the benefits of RDC. Obviously, our primary goal is to reduce retransmissions with and without RDC enabled. To this end, we consider weak RTT estimation, backoffs with variability, and dithering.

### 5.1. RTT Estimation

As shown in Figure 3, successful delivery without retransmission may be rare depending on the network condition. In such a case, it is useful to employ weak RTT estimation, which measures RTT using retransmitted segments. As mentioned in Section 3, CoCoA measures RTT between sending the first transmission and receiving the ACK, and it ignores RTT samples obtained after the third retransmission in order to avoid taking a much larger RTT estimate than the actual RTT. However, the results of our preliminary test with ContikiMAC indicated that we need to take a different approach to measuring weak RTT and setting RTO. We found that RTT sometimes got very large even when we took an RTT sample using the latest retransmission. In this case, measuring RTT between sending the first transmission and receiving the ACK would have rendered an RTT sample much larger than the actual RTT. Thus, we always measure RTT between the latest (re)transmission and the ACK regardless of how many retransmissions are performed. Once an RTT sample is taken, we calculate RTO without differentiating weak RTT from strong RTT.

### 5.2. Exponential Backoff with Variable Limits

We made a modification to uIP TCP so that the retransmission timer backs off based on the estimated RTO instead of the fixed RTO. Several studies argued that the mechanisms which use different backoff policies according to the RTO value are useful to avoid setting the retransmission timer to an excessively large value and to improve fairness between close nodes and distant nodes from the border router [1,19]. For this reason, CoCoA employs a variable backoff factor (VBF) mechanism which uses three backoff factors (3, 2, and 1.3) with two RTO thresholds of 1 and 3 s. 

However, when an RDC mechanism is enabled, RTO may be set to a very large value due to RTT fluctuation, as seen in the preliminary test result. This makes the CoCoA-like variable backoff factors unsuitable for uIP TCP in networks with an RDC mechanism enabled. Although we examined the effect of VBF mechanism where the thresholds were adapted to our network conditions, the result was not so good as expected. Thus, we consider varying the maximum number of backoffs according to RTO values. In choosing the maximum number of backoffs and the thresholds, we borrowed some numbers from the original uIP TCP. Recall that, in the original uIP TCP, the fixed RTO of 3 s backs off up to four times and, therefore, RTO increases up to 48 s (3 → 6 → 12 → 24 → 48). We simply chose the thresholds of 3, 6, and 12 s and then adjusted the maximum number of backoffs (4/3/2/1) so that RTO did not back off over 48 s. For example, if RTO is less than 3 s, RTO backs off up to four times. For RTO greater than 12 s, backoff is allowed only once. Table 2 shows the details.

### 5.3. Dithering

As discussed, it is important to adopt a measure to avoid synchronization like CoAP which dithers the retransmission timer value. We set the actual retransmission timer by adding a random duration to the retransmission timer. The interval for generating a random duration depends on the retransmission timer value. To avoid setting the actual retransmission timer to an excessively large value, we set the interval by multiplying the retransmission timer value by 1, 1/2, or 1/4 with the thresholds of 4 and 12 s. The reason for choosing the multipliers of 1, 1/2, and 1/4 is that the multiplication can be implemented using the shift operation. The thresholds were chosen empirically. For other kinds of networks, the multipliers and thresholds may be optimized according to the RTT distribution. It is important to note that, since uIP TCP uses the time granularity of 0.5 s for the retransmission timer, the effect of dithering is limited. Nevertheless, the simulation results show that even the limited dithering is effective in improving the performance of uIP TCP. 

Our algorithm to set the actual retransmission timer is summarized in Table 2.

## 6. Performance Evaluation

To evaluate the performance of our proposed scheme, we used the grid topologies as in the preliminary test. The maximum number of retransmissions was set to 15. We used three performance metrics: throughput (i.e., TCP goodput), total number of retransmissions, and fairness. Fairness between TCP flows is quantified using Jain’s index. We compared performance between three schemes: the original uIP TCP, the uIP TCP with dithering, and our proposed scheme. The uIP TCP with dithering was made by adding only dithering to the original uIP TCP. All simulations were run for 10 min and repeated 10 times. In the figures for simulation results, we added the 95% confidence intervals for the means in each plot.

**Figure 4:**
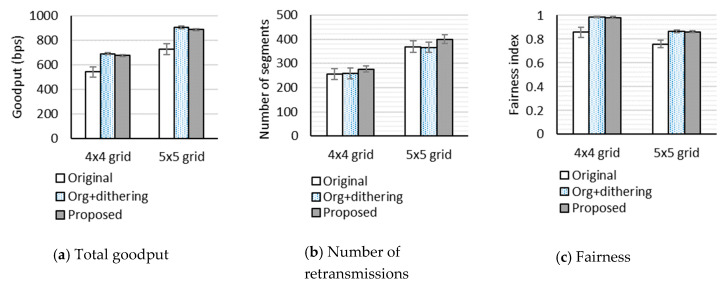
Performance in 4 × 4 grid and 5 × 5 grid topology networks when no RDC mechanism is used.

Firstly, to investigate the effect of the network size, we compared performance of the three schemes for the 4 × 4 grid and 5 × 5 grid topology networks. Compared to the 4 × 4 grid topology, the 5 × 5 grid topology not only causes larger differences of path lengths but also worsens the hidden terminal problem. In this test, we set the random loss rates to 0% and the number of packets in the link-layer queue to eight packets. Figure 4 shows the performance when an RDC mechanism is not enabled. In terms of total goodput, the original uIP TCP underperforms the other two schemes. The uIP TCP with dithering and our proposed scheme show almost equal performance, but the uIP TCP with dithering performs slightly better, which indicates the benefits of dithering. The reason why the goodput difference between three schemes is not extremely large is that RTT is very small. In terms of the number of retransmitted segments, our proposed scheme underperforms slightly. The schemes using the fixed RTO of 3 s benefit from very small RTT and the random loss rate of 0%. The fairness index of the original uIP TCP is lower than that of the other two schemes. The fairness index of our proposed scheme is almost the same as that of the uIP TCP with dithering.

**Figure 5:**
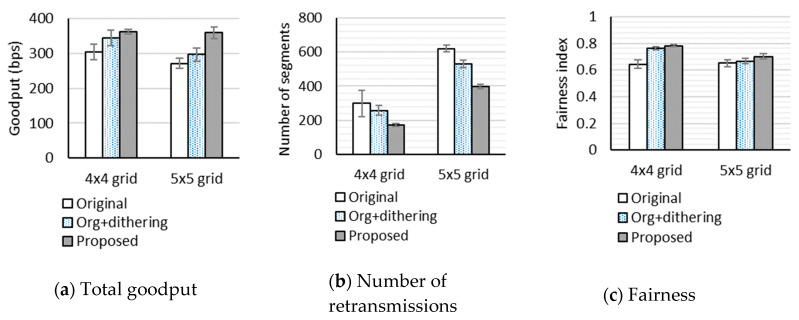
Performance in 4 × 4 grid and 5 × 5 grid topology networks when ContikiMAC is used.

Figure 5 shows the performance when ContikiMAC is used. For all schemes, the total goodput is much lower than when no RDC mechanism is used. The reason is that, as mentioned in Section 4.2.1, ContikiMAC worsens the hidden terminal problem. We note that the number of retransmissions of the original uIP TCP in the 5 × 5 grid topology network is very large. It is even larger than the number of successfully received segments. In Figure 5a,b, the 95% confidence intervals for the means of our proposed scheme do not overlap those of the original uIP TCP for both topologies. This suggests that our proposed scheme significantly outperforms the original uIP TCP in terms of goodput and the number of retransmissions, because it can adapt the actual retransmission timer to the large RTT fluctuations. Moreover, we observe from Figure 5a,b that, for the 5 × 5 grid topology, the 95% confidence intervals for the means of our proposed scheme and the uIP TCP with dithering do not overlap. This implies that a more severe hidden terminal problem results in a more effective retransmission timer adjustment based on RTT estimates. It is notable that, when ContikiMAC is used, TCP connection failures are often observed. In particular, the original uIP TCP suffers from more TCP connection failures than the others, resulting in a lower fairness index.

**Figure 6:**
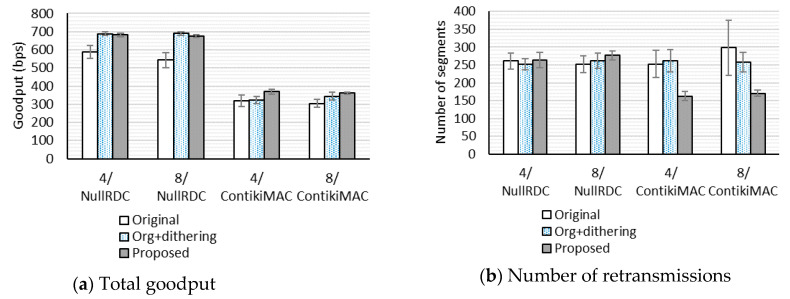
Effects of the link-layer queue size.

To investigate the impact of the queue size, we compare performance with the number of packets in the link-layer queue set to four packets and eight packets in the 4 × 4 grid topology network. As shown in Figure 6, the increase in the link-layer queue size from four packets to eight packets does not significantly impact goodput and the number of retransmissions. In Figure 6a, there is no overlap of the 95% confidence intervals for the means between the original uIP TCP and our proposed scheme, which shows that our proposed scheme can improve goodput for both queue sizes. Figure 6b shows that, when ContikiMAC is used, the 95% confidence intervals for the means of the original uIP TCP are relatively large, but do not overlap those of our proposed scheme. This implies that, when ContikiMAC is used, our proposed scheme reduces retransmissions for both queue sizes.

**Figure 7:**
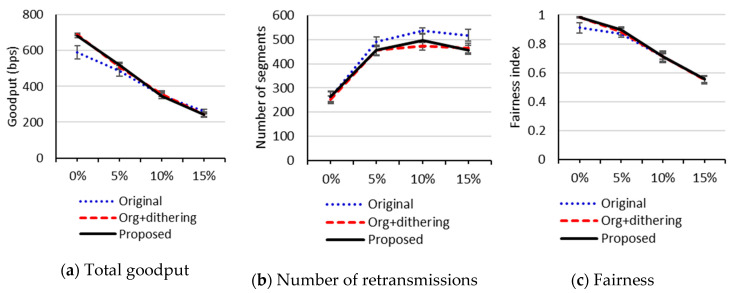
Performance with respect to random loss rates in a 4 × 4 grid topology network when an RDC mechanism is not enabled.

**Figure 8:**
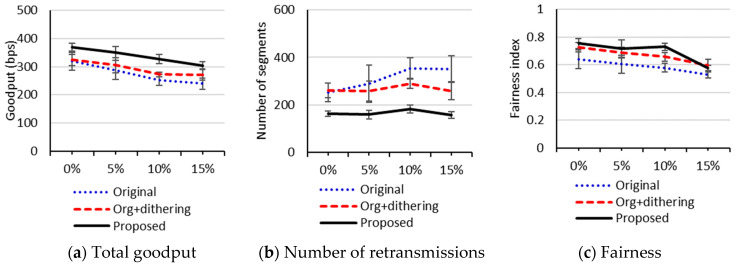
Performance with respect to random loss rates in a 4 × 4 grid topology network when ContikiMAC is used.

To figure out the impact of the random loss rates, we compared performance between the three schemes with the random loss rates of 0%, 5%, 10%, and 15%. In this test, the number of packets in the link layer queue was set to 4. We used the 4 × 4 grid topology because too many connection failures occurred in the 5 × 5 grid topology. Figure 7 depicts the performance when no RDC mechanism is used. As the random loss rate increases, the total goodputs and the fairness indexes of all the schemes converge. However, in Figure 7b, showing the number of retransmissions, the 95% confidence intervals for the means of our proposed scheme do not overlap those of the original uIP TCP with the loss rates of 10% and 15%. This implies that our proposed scheme is effective for reducing the number of retransmissions with high loss rates. Figure 8 shows the performance when ContikiMAC is used. We can see that, for all the schemes, the performance is less sensitive to random loss rates than when no RDC mechanism is used. In Figure 8a,b, the 95% confidence intervals for the means of the original uIP TCP do not overlap those of our proposed scheme at all loss rates of 0% through 15%, which indicates that our proposed scheme significantly improves goodput and the number of retransmissions. However, we observe from Figure 8c, depicting fairness, that there are overlaps between the confidence intervals of the original uIP TCP and our proposed scheme at the loss rates of 5% and 15%. As the random loss rate increases, the frequency of connection failure also tends to increase for all three protocols, which remains as an issue to be addressed in the future work.

## 7. Conclusions

uIP TCP may suffer lots of retransmissions and low throughput when RTT fluctuates wildly due to the hidden terminal problem, which may worsen with RDC enabled. One of the main reasons for such poor performance is that uIP TCP sets the retransmission timer using the fixed RTO for retransmitted segments. To address this problem, we propose a new scheme for retransmission timer management. Our scheme adjusts the retransmission timer according to the estimated RTT for retransmitted segments, as well as first-transmitted segments. In addition, our scheme adopts the notion of weak RTT estimation of CoCoA, exponential backoffs with variable limits, and dithering. Simulation results show that our proposed scheme enhances throughput and significantly reduces retransmissions, particularly when an RDC mechanism is enabled. 

In this work, we only used a periodic reporting scenario without bursty background traffic. For future work, we plan to extend our scheme considering various scenarios. Furthermore, we will examine the effect of extending the TCP window size to more than one segment. 

## Figures and Tables

**Table 1:** Simulation parameters.

Parameters	Values
Radio model	Unit Disk Graph Medium (UDGM)
Random loss rate	0%, 5%, 10%, or 15%
Transmission/interference range	50 m/100 m
Radio duty cycling mechanism	NullRDC or ContikiMAC (RDC channel check rate: 8 Hz)
RPL objective function	MRHOF (Minimum Rank with Hysteresis Objective Function)
Number of packets in the link-layer queue	4 or 8
Length of TCP payload	48 bytes
Offered load per node	64 bps
Simulation duration	10 min

**Table 2:** Proposed algorithm.

Event	Action
Initiation	MDEV = 16 s
An RTT sample is taken from the latest transmission	Err = RTT − SRTT SRTT = SRTT + 0.125 × Err MDEV = MDEV + 0.25 * (|Err| − MDEV) RTO = SRTT + 4 × MDEV retransmission_timer = RTO // dithering If (retransmission_timer > 4 s) retransmission_timer += random integer in [0, retransmission_timer × 0.5) else retransmission_timer += random integer in [0, retransmission_timer)
Retransmission timer expires	// variable limits on the maximum backoffs If RTO ≤ 3 s then n_backoff = max (#retransmissions, 4) else if RTO ≤ 6 s then n_backoff = max (#retransmissions, 3) else if RTO ≤ 12 s then n_backoff = max (#retransmissions, 2) else n_backoff = max (#retransmissions, 1) retransmission_timer = max(RTO << n_backoff, 48 s) // dithering If (retransmission_timer > 12 s) retransmission_timer += random integer in [0, retransmission_timer × 0.25) else if (retransmission_timer > 4 s) retransmission_timer += random integer in [0, retransmission_timer × 0.5) else retransmission_timer += random integer in [0, retransmission_timer]
